# Translational Research in Peripheral Nerve Repair and Regeneration

**DOI:** 10.1155/2014/381426

**Published:** 2014-09-09

**Authors:** Nektarios Sinis, Stefano Geuna, Fausto Viterbo

**Affiliations:** ^1^Department of Plastic, Hand, and Reconstructive Microsurgery, St. Marien Hospital Berlin, 12249 Berlin, Germany; ^2^Department of Clinical and Biological Sciences and Neuroscience Institute of the “Cavalieri Ottolenghi” Foundation (NICO), University of Turin, 10156 Orbassano, Italy; ^3^Clinic for Plastic Surgery, Dr. Fausto Viterbo, Rua Domingos Minicucci Filho, 587 Botucatu, SP, Brazil

The interest towards research in peripheral nerve repair regeneration has seen a great and progressive increase in the light of the continuous increase in the number of microsurgical nerve reconstructions worldwide [1]. While only few years ago nerve reconstruction was a rare type of surgery carried out in few high specialized centers only, it is becoming more and more frequent and widespread today and, considering the great number of traumatic events which could benefit from such type of surgery, this increasing tendency is going to continue in the next future making complex surgical interventions (such as limb replantation and brachial plexus reconstruction) more and more common worldwide.

In spite of the progress in the surgical techniques, it is getting more and more clear that peripheral nerve repair is no longer a matter of surgical reconstruction only but rather a matter of multidisciplinary and integrated treatment strategies [2].

This special issue brings together 11 reviews and research articles which nicely illustrate the multidisciplinary approach to translational research in peripheral nerve repair and regeneration.

While some cutting-edge surgical strategies, such as end-to-side neurorrhaphy [3], synthetic tubulization [4], and muscle neurotization [5], are being introduced to the clinical practice based on solid preclinical studies, basic research is exploring innovative approaches along four main lines.

First, cell transplantation is being explored with the goal to get information about the homing of transplanted cells in the receiving nerve as well as to identify which cell type best adapts to the nerve environment [6].

Second, various new types of biomimetic biomaterials are being tested as peripheral nerve scaffolds with a special view not only on the chemical composition of the material but also on its 3D configuration which might facilitate the regrowth of axons and the migration of Schwann cells [7].

Third, a large body of basic research is also dedicated to the identification of new molecules that might promote nerve regeneration, for example, inflammatory cytokines [8] and gliotrophic factors [9], as well as their controlled local release.

Last but not least, the possibility to promote nerve regeneration by physical therapy, that is, the application of physical agents, is also raising much interest among basic scientists [10].

The collection of papers published in this special issue nicely covers all these fields of study and we hope that it will attract the interests of a large number of interdisciplinary scientists as well as surgeons promoting successful translational research aimed at improving the treatment of peripheral nerve injury.

*Nektarios Sinis* *Stefano Geuna* *Fausto Viterbo*
